# Muscle mass loss is associated with physical dysfunction in patients with early rheumatoid arthritis

**DOI:** 10.3389/fnut.2022.1007184

**Published:** 2022-11-23

**Authors:** Jie Pan, Yao-Wei Zou, Ying-Ying Zhu, Jian-Zi Lin, Tao Wu, Ze-Hong Yang, Xue-Pei Zhang, Qian Zhang, Hu-Wei Zheng, Xiao-Ling He, Wan-Mei Cheng, Jian-Da Ma, Lie Dai

**Affiliations:** ^1^Department of Rheumatology, Sun Yat-Sen Memorial Hospital, Sun Yat-sen University, Guangzhou, China; ^2^Division of Clinical Research Design, Sun Yat-Sen Memorial Hospital, Sun Yat-sen University, Guangzhou, China; ^3^Department of Radiology, Sun Yat-Sen Memorial Hospital, Sun Yat-sen University, Guangzhou, China; ^4^Shanghai Healthcare Co. Ltd., Zhangjiang Innopark, Shanghai, China

**Keywords:** early rheumatoid arthritis, body composition, muscle mass loss, physical dysfunction, inflammation

## Abstract

**Background:**

Muscle mass loss is common in long-standing rheumatoid arthritis (RA). The aim was to explore the prevalence and effects of RA disease characteristics in patients with early RA.

**Methods:**

This cross-sectional study was carried out based on a Chinese RA cohort and control subjects. The body composition (BC) was assessed using bioelectric impedance analysis. Myopenia was defined by an appendicular skeletal muscle mass index of ≤ 7.0 kg/m^2^ in men and ≤ 5.7 kg/m^2^ in women. Physical dysfunction was defined as a health assessment questionnaire disability index > 1. Propensity score matching was performed to balance age and gender differences among patients with early RA (disease duration ≤ 12 months) and established RA, and controls (with 1:3:3 matching).

**Results:**

In total, 2017 controls and 1,008 patients with RA were recruited for this study. Among the patients with RA, there were 190 (18.8%) patients with early RA, with a median disease duration of 7 (4, 11) months. The matched patients with early RA (*n* = 160) showed a higher prevalence of myopenia than the matched controls (41.3 vs. 15.8%, *P* < 0.0167), but no difference was found in the matched patients with established RA (41.3 vs. 50.4%, *P* > 0.0167). Compared with the patients with established RA, the patients with early RA exhibited higher disease activity scores [disease activity score in 28 joints with four variables including C-reactive protein (DAS28-CRP): median 4.76 vs. 3.93, *P* < 0.001] and a higher prevalence of physical dysfunction (26.3 vs. 19.4%, *P* = 0.035). In the patients with early RA, patients with myopenia showed a higher prevalence of physical dysfunction than those without myopenia (41.3 vs. 15.5%, *P* < 0.001), among which walking and common daily activities were the most involved subdimensions. Multivariate logistic regression analysis showed that DAS28-CRP was positively associated with myopenia [adjusted odds ratio (AOR) 1.558, 95% CI (1.138–2.132)], and myopenia [AOR 2.983, 95% CI (1.192–7.465)] was independently associated with physical dysfunction in the patients with early RA.

**Conclusion:**

Our data indicate the importance of early detection of muscle involvement in the early stage of RA and imply the significance of early aggressive control of disease activity for the prevention of myopenia and physical dysfunction in patients with early RA. Our study provides a new perspective on RA management.

## Introduction

Rheumatoid arthritis (RA) is a common chronic inflammatory disease leading to joint damage and disability (1). An unfavorable alteration of body composition (BC) has been reported in patients with RA, including a loss of muscle mass, concurrent with or without increased fat mass (2–6). Myopenia, a novel nomenclature for the presence of muscle wasting caused by clinical diseases, has been reported to be common in patients with RA (7). We previously reported that 45.1% of Chinese patients with RA were complicated with myopenia (3), especially elderly patients with RA (54.5%) (4). The increasing prevalence of myopenia has been well-recognized in patients with long-standing RA, which may be associated with prolonged disease duration (3, 4, 8). However, less is known about BC alteration and its effects on RA disease characteristics in the early stage of RA.

Myopenia has been reported to be associated with more active disease, worsening physical dysfunction, severe joint destruction, and even increased risk of cardiovascular diseases and mortality in patients with established RA (3, 4, 8). However, the results of the associations between RA disease characteristics and loss of muscle mass in patients with early RA are inconsistent regarding the restricted evidence. Furthermore, there is a lack of comparison of muscle mass and strength between patients with early and established RA. Based on our large-scale RA cohort, we aimed to explore the alteration and significance of BC in patients with early RA with a disease duration ≤ 12 months and to compare with matched controls as well as patients with established RA.

## Methods

### Study population

We conducted a cross-sectional study which was drawn from our RA cohort (3, 4). Patients in the hospital ward or outpatient clinic who fulfilled the 2010 classification criteria for RA (9) were recruited from September 2015 to January 2022. Patients younger than 16 years and those accompanied by other connective tissue diseases, infection, malignancy, pregnancy, and other diseases that may affect physical function (e.g., stroke) were excluded. According to disease duration, patients were classified as early ( ≤ 12 months) or established RA (>12 months) (10). Controls were staff in Zhangjiang InnoPark of Shanghai from June 2015 to August 2016 and in Sun Yat-Sen Memorial Hospital from May 2019 to March 2022 who voluntarily participated in this study. These control subjects were excluded if self-reported with a history of connective tissue diseases, cancer, severe chronic diseases, mental disorders, implanted electronic devices, current infection, or pregnancy.

### Demographic and clinical data collection

The demographic and clinical data of patients with RA were recorded at enrollment, as we previously reported (3, 4), including gender, age, smoking history, body mass index (BMI), disease duration, disease activity, radiographic indicators, physical function, comorbidities, serum albumin, and previous medications. Disease activity was assessed with a disease activity score in 28 joints with four variables including erythrocyte sedimentation rate (DAS28-ESR), disease activity score in 28 joints with four variables including C-reactive protein (DAS28-CRP), Simplified Disease Activity Index (SDAI), and Clinical Disease Activity Index (CDAI). Radiographic indicators included modified total Sharp score (mTSS), joint space narrowing (JSN) subscore, and joint erosion (JE) subscore.

Physical function was measured by the Stanford Health Assessment Questionnaire (HAQ). HAQ is a disease-specific instrument to assess the functional status of RA, which includes eight subdimensions (11). The HAQ score ranges from 0 to 3, with higher scores indicating more severe dysfunction. Physical dysfunction was defined as a HAQ disability index (HAQ-DI) > 1, as described previously (3, 12), and physical dysfunction of the eight subdimensions was defined as the score of that subdimension > 1.

### Body composition assessments

Body composition parameters were measured by bioelectric impedance analysis using an InBody 230 device (Biospace Co., Shanghai, China), as we previously reported (3, 4). The appendicular skeletal muscle mass index (ASMI) was defined as appendicular skeletal muscle mass/height^2^ (kg/m^2^). Myopenia was defined by an ASMI ≤ 7.0 kg/m^2^ in men and ≤ 5.7 kg/m^2^ in women (13), while overfat was defined by the percentage of body fat mass (BF%) ≥ 25% in men and ≥ 35% in women, respectively (14).

### Statistical analysis

Statistical analyses were conducted by SPSS software (version 25.0) and R software (version 4.0.4). Differences between the two groups were detected by Student's *t*-test or the Mann–Whitney *U*-test or chi-squared test for continuous or categorical variables. Propensity score matching was brought to balance age and gender distribution among the patients with early RA and established RA, and controls (in 1:3:3 matching) for comparisons of BC characteristics. One-way analysis of variance (ANOVA) and the chi-squared test were used for the comparison of continuous variables and categorical variables among the three groups, respectively. Bonferroni correction was further performed for multiple comparisons to correct the type I error. Subgroup analyses according to gender were conducted to estimate the differences in BC characteristics among the three groups.

Logistic regression analyses were conducted to evaluate the associations between RA disease activity (including DAS28-ESR, DAS28-CRP, SDAI, and CDAI) and myopenia/overfat in the patients with early and established RA separately. In Model 1, gender, age, smoking history, rheumatoid factor (RF) status, anti-cyclic citrullinated peptide antibody (ACPA) status, comorbidities, level of serum albumin, and previous treatment were adjusted for, while the mTSS was further added to Model 2, and BMI and BF%/ASMI were added to Model 3. Similar logistic regression analyses in different adjusting models were performed to identify the associations between BMI, BC (ASMI, myopenia, BF%, and overfat), and physical dysfunction in the patients with early and established RA separately. Subgroup analyses according to gender were also performed to distinguish whether there were differences in the associations of BC with physical dysfunction between female and male patients with early and established RA. A two-tailed *P*-value < 0.05 was considered statistically significant, except for the *P*-value (< 0.0167) of Bonferroni correction used in multiple comparisons.

## Results

### Demographic and clinical characteristics of patients with RA

**Figure 1:**
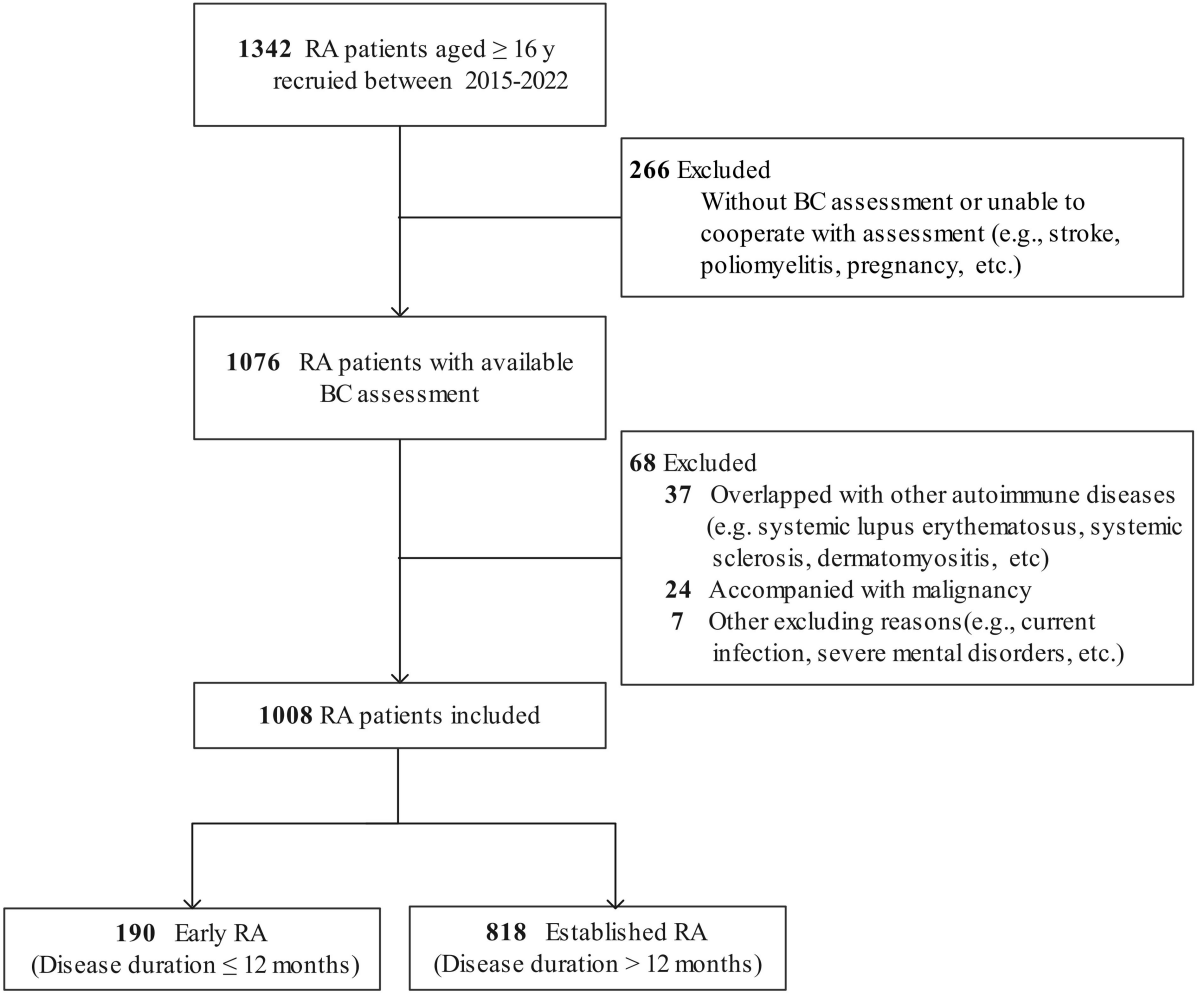
Flow diagram of patients with RA included. RA, rheumatoid arthritis; BC, body composition.

Of the 1,342 patients with RA recruited, 266 patients without BC assessment and 68 patients with other diseases were excluded. Hence, a total of 1,008 patients with RA and 2017 controls were qualified for analysis (Figure 1). In the RA group, 809 (80.3%) patients were female, with a mean age of 51.1 ± 12.6 years and a median disease duration of 57 (IQR 21–120) months. According to CDAI, 184 (18.3%) patients were in remission (CDAI ≤ 2.8), while 824 (81.7%) were active (CDAI > 2.8). In total, 21.9% of patients with RA were treatment-naive who had not received glucocorticoids or disease-modifying antirheumatic drug (DMARD) therapy for at least 6 months before enrollment (Supplementary Table 1).

There were 190 (18.8%) patients with early RA (median disease duration 7 months) and 818 (81.2%) patients with established RA (median disease duration 72 months). Compared with the patients with established RA, the patients with early RA were younger (mean 49.2 years vs. 51.6 years), had a higher proportion of male patients (28.4 vs. 17.7%), and had a shorter disease duration and lower radiographic scores (all *P* < 0.05). Meanwhile, the patients with early RA had a lower level of serum albumin, more active RA, and more severe and higher prevalence of physical dysfunction (all *P* < 0.05, Supplementary Table 1).

### BC characteristics of patients with early RA and established RA

Of the control subjects, there were 53.0% female subjects, with a mean age of 34.8 ± 10.1 years. Compared with the controls, the patients with RA were more predominantly female and older (both *P* < 0.001). After matching by gender and age (1:3:3), 160 patients with early RA, 480 patients with established RA, and 480 controls were qualified for comparison.

Compared with the matched controls, both the matched patients with early and established RA had lower BMI, decreased skeletal muscle mass and muscle mass distributed in the trunk and appendicular extremities, together with a higher prevalence of myopenia (early RA: 41.3 vs. 15.8%; established RA: 50.4 vs. 15.8%, both *P* < 0.0167, Table 1). Although the patients with established RA showed further decreased muscle mass, the prevalence of myopenia in patients with early RA was as high as that in established RA (41.3 vs. 50.4%, *P* > 0.0167). For body fat mass, when compared with the matched controls, the patients with established RA, but not early RA, showed a higher prevalence of overfat (established RA: 32.7 vs. 22.7%, *P* < 0.0167; early RA: 26.9 vs. 22.7%, *P* > 0.0167).

**Table 1:** Comparisons of BC characteristics among patients with early RA, established RA, and controls.

**Characteristics**	**Before matching**				After matching*			
	**Control subjects (*n* = 2,017)**	**Early RA (*n* = 190)**	**Established RA (*n* = 818)**	***P***	**Control subjects (*n* = 480)**	**Early RA (*n* = 160)**	**Established RA (*n* = 480)**	***P***
Female, *n* (%)	1,069 (53.0)	136 (71.6)	673 (82.3)	**< 0.001**	357 (74.4)	108 (67.5)	346 (72.1)	0.236
Age, years	34.8 ± 10.1	49.2 ± 13.4^#^	51.6 ± 12.4^#&^	**< 0.001**	46.2 ± 10.2	46.8 ± 12.2	46.6 ± 12.3	0.782
Height, cm	166.5 ± 8.1	160.5 ± 7.3^#^	157.7 ± 6.9^#&^	**< 0.001**	162.2 ± 7.4	161.0 ± 7.1	159.4 ± 7.1^#&^	**< 0.001**
Weight, kg	63.0 ± 12.3	56.1 ± 9.1^#^	54.5 ± 9.7^#^	**< 0.001**	61.0 ± 10.9	56.3 ± 8.8^#^	54.9 ± 9.7^#^	**< 0.001**
BMI, kg/m^2^	23.0 ± 3.2	21.8 ± 3.3^#^	21.9 ± 3.3^#^	**< 0.001**	23.1 ± 3.1	21.7 ± 3.2^#^	21.6 ± 3.2^#^	**< 0.001**
BMI subgroups				**< 0.001**				**< 0.001**
Underweight, *n* (%)	156 (7.7)	32 (16.8)^#^	126 (15.4)^#^		25 (5.2)	28 (17.5)^#^	87 (18.1)^#^	
Normal weight, *n* (%)	1,244 (61.7)	115 (60.5)	483 (59.0)		277 (57.7)	99 (61.9)	290 (60.4)	
Overweight, *n* (%)	514 (25.5)	37 (19.5)	172 (21.0)		152 (31.7)	28 (17.5)^#^	83 (17.3) ^#^	
Obese, *n* (%)	103 (5.1)	6 (3.2)	37 (4.5)		26 (5.4)	5 (3.1)	20 (4.2)	
**Muscle mass assessment**								
Skeletal muscle mass, kg	26.1 ± 6.0	21.7 ± 3.9^#^	20.2 ± 4.0^#&^	**< 0.001**	24.0 ± 5.3	22.0 ± 4.0^#^	20.9 ± 4.4^#&^	**< 0.001**
Trunk muscle, kg	20.8 ± 4.4	17.6 ± 3.1^#^	16.5 ± 3.2^#&^	**< 0.001**	19.5 ± 3.9	17.9 ± 3.1^#^	17.0 ± 3.5^#&^	**< 0.001**
Upper extremity muscle, kg	4.8 ± 1.4	3.9 ± 1.0^#^	3.5 ± 1.1^#&^	**< 0.001**	4.4 ± 1.3	3.9 ± 1.0^#^	3.7 ± 1.1^#&^	**< 0.001**
Lower extremity muscle, kg	15.0 ± 3.4	12.3 ± 2.5^#^	11.3 ± 2.5^#&^	**< 0.001**	13.5 ± 3.0	12.5 ± 2.5^#^	11.8 ± 2.6^#&^	**< 0.001**
ASMI, kg/m^2^	7.0 ± 1.1	6.2 ± 0.9^#^	5.9 ± 1.0^#&^	**< 0.001**	6.7 ± 1.1	6.3 ± 0.9^#^	6.0 ± 1.0^#&^	**< 0.001**
Myopenia, *n* (%)	278 (13.8)	80 (42.1) ^#^	420 (51.3)^#^	**< 0.001**	76 (15.8)	66 (41.3) ^#^	242 (50.4) ^#^	**< 0.001**
**Fat mass assessment**								
Fat mass, kg	15.8 ± 5.4	16.0 ± 6.4	16.8 ± 6.3^#^	**< 0.001**	17.2 ± 5.3	15.6 ± 6.2^#^	16.1 ± 6.1^#^	**0.003**
Trunk fat, kg	7.8 ± 3.0	7.7 ± 3.5	8.2 ± 3.4^#^	**0.015**	8.6 ± 3.0	7.6 ± 3.4^#^	7.8 ± 3.4^#^	**< 0.001**
Upper extremity fat, kg	2.0 ± 0.9	2.2 ± 1.1^#^	2.4 ± 1.1^#&^	**0.016**	2.2 ± 0.9	2.1 ± 1.0	2.2 ± 1.0	0.329
Lower extremity fat, kg	5.0 ± 1.5	5.0 ± 1.8	5.3 ± 1.8^#^	**< 0.001**	5.3 ± 1.4	4.9 ± 1.7^#^	5.1 ± 1.7	**0.014**
BF%, %	25.0 ± 6.6	27.9 ± 8.6^#^	30.3 ± 8.3^#^	**< 0.001**	28.0 ± 6.6	27.3 ± 8.7	28.9 ± 8.3	**0.044**
Overfat, *n* (%)	389 (19.3)	51 (26.8)^#^	294 (35.9)^#^	**< 0.001**	109 (22.7)	43 (26.9)	157 (32.7)^#^	**0.002**

Values of continuous variables were presented as mean and standard deviation or median with interquartile range according to distributions, and categorical variables were presented as numbers and percentages.

^*^Propensity score matching among patients with early RA and established RA, and controls with 1:3:3 matching by gender and age.

Comparisons among three groups by the one-way ANOVA test or chi-squared test. A P-value < 0.05 was defined as a significant difference, which was shown in bold.

^#^Compared patients in early and established RA groups with controls in Bonferroni correction, P < 0.0167.

^&^Compared patients in established RA with patients in early RA in Bonferroni correction, P < 0.0167.

BC, body composition; RA, rheumatoid arthritis; BMI, body mass index; ASMI, appendicular skeletal muscle mass index; BF%, percentage of body fat mass.

### Clinical characteristics of patients with myopenia or overfat in early and established RA

There were 42.1% (80/190) patients with early RA and 51.3% (420/818) patients with established RA had myopenia (*P* = 0.022). In the patients with early RA, the prevalence of myopenia was 37.5% (6/16) in patients with remission and 42.5% (74/174) in those with active RA, including 34.0% (17/50), 39.0% (23/59), and 52.3% (34/65) in patients with low disease activity (LDA, 2.8 < CDAI ≤ 10), moderate disease activity (MDA, 10 < CDAI ≤ 22), and high disease activity (HAD, CDAI > 22), respectively. While in the patients with established RA, the prevalence of myopenia was 40.5% (68/168) in patients with remission and 54.2% (352/650) in those with active RA (*P* = 0.697), including 44.3% (101/228), 56.2% (127/226), and 63.3% (124/196) with LDA, MDA, and HDA, respectively. Compared with those without myopenia, patients with myopenia in both early and established RA groups had higher levels of ESR and CRP, more active RA, a higher prevalence of cardiovascular diseases, and a lower level of serum albumin (Table 2). In addition, the patients with myopenia in the established RA group also had a longer disease duration and more severe radiographic damage including higher mTSS, JSN, and JE subscores than those without (all *P* < 0.05, Table 2).

**Table 2:** Clinical characteristics of patients with myopenia in early and established RA.

	**Early RA**			**Established RA**		
**Characteristics**	**Non-myopenia (*n* = 110)**	**Myopenia (*n* = 80)**	***P^#^***	**Non-myopenia (*n* = 398)**	**Myopenia (*n* = 420)**	***P^#^***
Female, *n* (%)	84 (76.5)	52 (65.0)	0.086	318 (79.9)	355 (84.5)	0.083
Age, years	48.3 ± 11.7	50.3 ± 15.5	0.225	51.5 ± 11.1	51.7 ± 13.5	0.211
Disease duration, month	8 (4, 11)	7 (4, 10)	0.605	60 (36, 120)	84 (36, 140)	**0.001**
Smoking history			**0.038**			0.984
Active smoking, *n* (%)	20 (18.2)	24 (30.0)		55 (13.8)	59 (14.0)	
Passive smoking, *n* (%)	35 (31.8)	14 (17.5)		114 (28.6)	118 (28.1)	
No smoking, *n* (%)	55 (50.0)	42 (22.1)		229 (57.5)	243 (57.9)	
Positive RF, *n* (%)	80 (72.7)	61 (76.3)	0.584	248 (62.3)	298 (71.0)	**0.009**
Positive ACPA, *n* (%)	87 (79.1)	59 (73.8)	0.389	273 (68.6)	305 (72.6)	0.206
**Core disease activity indicators**						
28TJC	4 (1, 8)	5 (2, 12)	0.112	2 (0, 5)	3 (1, 8)	**< 0.001**
28SJC	3 (0, 5)	3 (1, 9)	0.040	1 (0, 3)	2 (0, 5)	**< 0.001**
PtGA	4 (2, 6)	5 (2, 6)	**0.031**	3 (1, 5)	4 (2, 6)	**< 0.001**
PrGA	4 (2, 5)	5 (2, 6)	**0.022**	2 (1, 5)	4 (2, 6)	**< 0.001**
PainVAS	4 (2, 4)	4 (2, 6)	**0.013**	2 (1, 4)	3 (2, 5)	**< 0.001**
ESR (mm/h)	32 (18, 53)	53 (20, 89)	**0.008**	26 (14, 42)	35 (18, 68)	**< 0.001**
CRP (mg/L)	5.58 (3.21, 20.65)	20.10 (3.53, 62.95)	**0.001**	3.45 (3.23, 10.65)	6.13 (3.28, 23.65)	**< 0.001**
DAS28-ESR	4.66 (3.30, 5.52)	5.14 (3.69, 6.62)	**0.011**	3.55 (2.47, 4.76)	4.27 (3.06, 5.79)	**< 0.001**
DAS28-CRP	3.38 (2.51, 4.49)	4.12 (2.92, 5.24)	**0.006**	2.67 (1.74, 3.68)	3.29 (2.31, 4.58)	**< 0.001**
SDAI	14.73 (7.90, 25.82)	22.86 (10.52, 39.27)	**0.008**	9.41 (3.31, 18.31)	14.86 (6.31, 27.22)	**< 0.001**
CDAI	14 (7, 23)	19 (10, 34)	**0.031**	9 (2, 16)	14 (6, 24)	**< 0.001**
**Radiographic assessment**						
mTSS	2 (0, 5)	4 (0, 9)	0.135	8 (3, 25)	23 (8, 64)	**< 0.001**
JSN	0 (0, 1)	0 (0, 2)	0.071	2 (0, 10)	10 (2, 31)	**< 0.001**
JE	1 (0, 4)	2 (0, 7)	0.182	6 (2, 16)	13 (5, 34)	**< 0.001**
**Comorbidities**						
Hypertension, *n* (%)	30 (27.3)	18 (22.5)	0.455	144 (36.2)	129 (30.7)	0.097
Diabetes, *n* (%)	10 (9.1)	7 (8.8)	0.935	49 (12.3)	50 (11.9)	0.858
Dyslipidemia, *n* (%)	39 (35.5)	30 (37.5)	0.772	114 (28.6)	143 (34.0)	0.096
Cardiovascular diseases, *n* (%)	5 (4.5)	10 (12.5)	**0.045**	38 (9.5)	59 (14.0)	**0.047**
**Nutritional indicator**						
Serum albumin (g/L)	35.0 ± 3.8	32.3 ± 5.6	**< 0.001**	35.5 ± 4.3	33.9 ± 4.6	**< 0.001**
**Previous medications**						
Treatment-naive, *n* (%)	41 (37.3)	25 (31.3)	0.389	71 (17.8)	84 (20.0)	0.431
Glucocorticoids, *n* (%)	53 (48.2)	45 (56.3)	0.272	204 (51.3)	231 (55.0)	0.283
csDMARDs, *n* (%)	59 (53.6)	42 (52.5)	0.877	306 (76.9)	306 (72.9)	0.185
bDMADRs/tsDMARDs, *n* (%)	5 (4.5)	6 (7.5)	0.389	34 (8.5)	31 (7.4)	0.539

Values of continuous variables were presented as mean and standard deviation or median with an interquartile range according to distributions, and categorical variables were presented as numbers and percentages.

^#^Comparisons by Student's t-test, chi-squared test, or Mann–Whitney U-test. A P-value < 0.05 was defined as a significant difference, which was shown in bold.

RA, rheumatoid arthritis; RF, rheumatoid factor; ACPA, anti-cyclic citrullinated peptide antibody; 28TJC, 28-joint tender joint counts; 28SJC, 28-joint swollen joint counts; PtGA, patient global assessment of disease activity; PrGA, provider global assessment of disease activity; Pain VAS, pain visual analog scale; ESR, erythrocyte sedimentation rate; CRP, C-reactive protein; DAS28-ESR, disease activity score in 28 joints with four variables including erythrocyte sedimentation rate; DAS28-CRP, disease activity score in 28 joints with four variables including C-reactive protein; SDAI, Simplified Disease Activity Index; CDAI, Clinical Disease Activity Index; mTSS, modified total Sharp score; JSN, joint space narrowing; JE, joint erosion; csDMARDs, conventional synthetic disease-modifying antirheumatic drugs; bDMARDs, biological disease-modifying antirheumatic drugs; tsDMARDs, target synthetic disease-modifying antirheumatic drugs.

There were 26.8% (51/190) patients with early RA and 35.9% (294/818) patients with established RA showing overfat (*P* = 0.017). In the patients with early RA, the prevalence of overfat was 31.3% (5/16) in the patients with remission and 26.4% (46/174) in those with active RA, including 20.0% (10/50), 23.7% (14/59), and 33.8% (22/65) in LDA, MDA, and HDA, respectively. While in patients with established RA, the prevalence of overfat was 29.8% (50/168) in the patients with remission and 37.5% (244/650) in those with active RA (*P* = 0.002), including 32.5% (74/228), 33.2% (75/226), and 48.5% (95/196) in LDA, MDA, and HDA, respectively. Compared with those without overfat, patients with overfat in both the early and established RA groups were older, had more severe radiographic damage including higher mTSS and JE subscores and had more comorbidities (all *P* < 0.05, Supplementary Table 2). Additionally, the patients with overfat in the established RA group were also characterized with higher levels of ESR and CRP, and more active RA than those without overfat (all *P* < 0.05, Supplementary Table 2).

### Associations of disease activity with myopenia or overfat in patients with early and established RA

**Figure 2:**
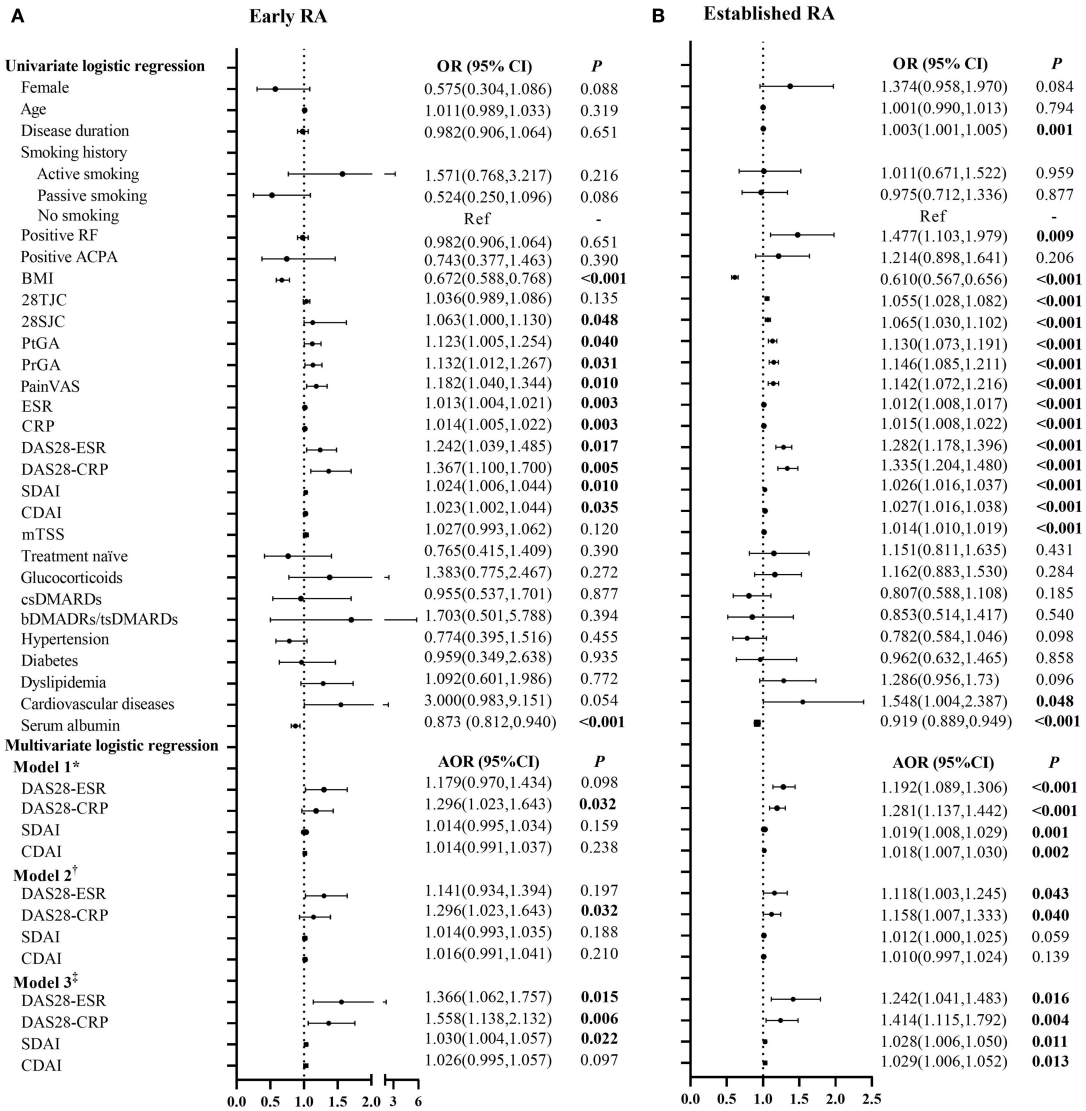
Logistic regression analysis of the relevant characteristics of myopenia in patients with early and established RA. *Model 1 adjusted for gender, age, smoking history, disease duration, RF status, ACPA status, comorbidities, serum albumin, and previous treatment. ^†^Model 2 adjusted for Model 1+ mTSS. ^‡^Model 3 adjusted for Model 2+BMI and BF%. OR, odds ratio in logistic regression; 95% CI, 95% confidence interval; RA, rheumatoid arthritis; RF, rheumatoid factor; ACPA, anti-cyclic citrullinated peptide antibody; BMI, body mass index; 28TJC, 28-joint tender joint counts; 28SJC, 28-joint swollen joint counts; PtGA, patient global assessment of disease activity; PrGA, provider global assessment of disease activity; Pain VAS, pain visual analog scale; ESR, erythrocyte sedimentation rate; CRP, C-reactive protein; DAS28-ESR, disease activity score in 28 joints with four variables including erythrocyte sedimentation rate; DAS28-CRP, disease activity score in 28 joints with four variables including C-reactive protein; SDAI, Simplified Disease Activity Index; CDAI, Clinical Disease Activity Index; mTSS, modified total Sharp score; csDMARDs, conventional synthetic disease-modifying antirheumatic drugs; bDMARDs, biological disease-modifying anti-rheumatic drugs; tsDMARDs, target synthetic disease-modifying antirheumatic drugs; BF%, percentage of body fat mass. **(A)** Early RA. **(B)** Established RA.

The associations of RA disease activity with myopenia or overfat in patients with early and established RA are represented in Figure 2 and Supplementary Table 3. Higher disease activity scores, including DAS28-ESR, DAS28-CRP, SDAI, and CDAI, were positively associated with myopenia in both the patients with early and established RA (all *P* < 0.05). Even after adjusting all other potential confounding factors by step (Model 1 to Model 3), there were still positive associations between DAS28-CRP and myopenia in both the patients with early [Model 3, adjusted odds ratio (AOR) 1.558, 95% CI (1.138–2.132), Figure 2A] and established RA [AOR 1.414, 95% CI (1.115–1.792), Figure 2B].

However, no significant association of disease activity scores with overfat was found in the patients with early RA. For the patients with established RA, higher disease activity scores were positively associated with overfat in univariate logistic regression, but the associations disappeared after adjustment for mTSS (Model 2), and further adding an adjustment for BMI and ASMI (Model 3, Supplementary Table 3).

### Clinical and BC characteristics in patients with early and established RA with physical dysfunction

There were 26.3% (50/190) patients with early RA and 19.4% (159/818) patients with established RA showing physical dysfunction (*P* = 0.035). The patients with physical dysfunction in both the early and established RA groups were older and had more active RA disease, more comorbidities (except hypertension in early RA), and a lower level of serum albumin compared with those without physical dysfunction. Contrary to the patients with physical dysfunction in the established RA group who had long-standing disease durations and higher radiographic scores, the patients with physical dysfunction in the early RA group had shorter disease durations and no significant difference in radiographic scores than those without physical dysfunction (Table 3). Importantly, the patients with physical dysfunction in both the early and established RA groups had lower levels of ASMI and higher proportions of myopenia and overfat than those without physical dysfunction. While only patients with physical dysfunction in the established RA group had a higher level of BF% than those without physical dysfunction (all *P* < 0.05, Table 3).

**Table 3:** Clinical and BC characteristics of patients with physical dysfunction in early and established RA.

	**Early RA**			**Established RA**		
**Characteristics**	**Without physical dysfunction (*n* = 140)**	**With physical dysfunction (*n* = 50)**	***P^#^***	**Without physical dysfunction (*n* = 659)**	**With physical dysfunction (*n* = 159)**	***P^#^***
Female, *n* (%)	103 (73.6)	33 (66.0)	0.308	546 (82.9)	127 (79.9)	0.377
Age, years	47.6 ± 12.1	53.4 ± 15.7	**0.003**	50.5 ± 12.4	56.2 ± 11.3	**< 0.001**
Disease duration, month	9 (5, 11)	6 (4, 9)	**0.014**	70 (36, 120)	117 (50, 180)	**< 0.001**
Smoking history			0.135	84 (12.7)	25 (15.7)	0.610
Active smoking, *n* (%)	29 (20.7)	15 (30.0)		88 (13.4)	26 (16.4)	
Passive smoking, *n* (%)	41 (29.3)	8 (16.0)		189 (28.7)	43 (27.0)	
No smoking, *n* (%)	70 (50.0)	27 (54.0)		382 (58.0)	90 (56.6)	
Positive RF, *n* (%)	104 (74.3)	37 (74.0)	0.968	424 (64.3)	122 (76.7)	**0.003**
Positive ACPA, *n* (%)	109 (77.9)	37 (74.0)	0.579	465 (70.6)	113 (71.1)	0.900
**Core disease activity indicators**						
28TJC	3 (1, 5)	12 (8, 15)	**< 0.001**	2 (0, 4)	10 (5, 16)	**< 0.001**
28SJC	2 (0, 4)	8 (5, 11)	**< 0.001**	1 (0, 2)	7 (3, 11)	**< 0.001**
PtGA	3 (2, 5)	7 (6, 8)	**< 0.001**	2 (1, 5)	7 (5, 8)	**< 0.001**
PrGA	3 (2, 4)	7 (6, 8)	**< 0.001**	2 (1, 4)	7 (5, 8)	**< 0.001**
PainVAS	2 (2, 4)	6 (4, 8)	**< 0.001**	2 (1, 4)	6 (4, 7)	**< 0.001**
ESR (mm/h)	30 (15, 53)	73 (51, 107)	**< 0.001**	26 (14, 44)	64 (37, 93)	**< 0.001**
CRP (mg/L)	5.17 (3.16, 20.40)	51.35 (16.73, 84.98)	**< 0.001**	3.30 (3.23, 10.90)	19.90 (5.81, 47.00)	**< 0.001**
DAS28-ESR	3.190 (2.40, 3.95)	5.19 (4.70, 5.76)	**< 0.001**	2.65 (1.77, 3.56)	4.92 (3.97, 5.59)	**< 0.001**
DAS28-CRP	4.20 (3.16, 4.98)	6.65 (6.07, 7.23)	**< 0.001**	3.50 (2.50, 4.52)	6.27 (5.39, 7.00)	**< 0.001**
SDAI	13.32 (6.48, 21.07)	39.27 (29.34, 48.73)	**< 0.001**	9.31 (3.31, 16.33)	33.20 (24.56, 44.34)	**< 0.001**
CDAI	12 (6, 19)	34 (26, 42)	**< 0.001**	8 (2, 15)	30 (22, 41)	**< 0.001**
**Functional indicators**						
HAQ-DI	0.13 (0.00, 0.50)	1.88 (1.38, 2.50)	**< 0.001**	0.13 (0.00, 0.50)	1.63 (1.38, 2.13)	**< 0.001**
**Radiographic assessment**						
mTSS	2 (0, 6)	4 (0, 9)	0.126	11 (4, 33)	42 (12, 127)	**< 0.001**
JSN	0 (0, 1)	0 (0, 2)	0.073	4 (0, 15)	15 (3, 44)	**< 0.001**
JE	1 (0, 5)	2 (0, 7)	0.186	8 (3, 20)	19 (7, 53)	**< 0.001**
**Comorbidities**						
Hypertension, *n* (%)	35 (25.0)	13 (26.0)	0.889	208 (31.6)	65 (40.9)	**0.025**
Diabetes, *n* (%)	8 (5.7)	9 (18.0)	**0.009**	67 (10.2)	32 (20.1)	**0.001**
Dyslipidemia, *n* (%)	45 (32.1)	24 (48.0)	**0.045**	187 (28.4)	70 (44.0)	**< 0.001**
Cardiovascular diseases, *n* (%)	7 (5.0)	8 (16.0)	**0.013**	60 (9.1)	37 (23.3)	**< 0.001**
**Nutritional indicator**						
Serum albumin (g/L)	34.8 ± 4.3	31.3 ± 5.2	**< 0.001**	35.2 ± 4.4	32.4 ± 4.6	**< 0.001**
**Previous medications**						
Treatment-naive, *n* (%)	41 (29.3)	25 (50.0)	**0.008**	89 (13.5)	66 (41.5)	**< 0.001**
Glucocorticoids, *n* (%)	80 (57.1)	18 (36.0)	**0.010**	356 (54.0)	79 (49.7)	0.325
csDMARDs, *n* (%)	83 (59.3)	18 (36.0)	**0.005**	544 (82.5)	68 (42.8)	**< 0.001**
bDMADRs/tsDMARDs, *n* (%)	8 (5.7)	3 (6.0)	0.100	56 (8.5)	9 (5.7)	0.235
**BC characteristics**						
BMI, kg/m^2^	21.9 ± 3.3	21.6 ± 3.1	0.575	21.9 ± 3.3	21.7 ± 3.3	0768
ASMI, kg/m^2^	6.3 ± 0.8	6.0 ± 1.1	**0.044**	6.0 ± 0.9	5.5 ± 1.1	**< 0.001**
Myopenia, *n* (%)	47 (33.6)	33 (66.0)	**< 0.001**	310 (47.0)	110 (69.2)	**< 0.001**
BF%, %	27.7 ± 8.4	28.6 ± 9.4	0.651	29.9 ± 8.0	32.3 ± 8.7	**0.001**
Overfat, *n* (%)	32 (22.9)	19 (38.0)	**0.038**	207 (31.4)	87 (54.7)	**< 0.001**

Values of continuous variables were presented as mean and standard deviation or median with interquartile range according to distributions, and categorical variables were presented as numbers and percentages.

^#^Comparisons by Student's t-test, chi-squared test, or Mann–Whitney U-test. A P-value < 0.05 was defined as significant differences, which was shown in bold.

BC, body composition; RA, rheumatoid arthritis; RF, rheumatoid factor; ACPA, anti-cyclic citrullinated peptide antibody; 28TJC, 28-joint tender joint counts; 28SJC, 28-joint swollen joint counts; PtGA, patient global assessment of disease activity; PrGA, provider global assessment of disease activity; Pain VAS, pain visual analog scale; ESR, erythrocyte sedimentation rate; CRP, C-reactive protein; DAS28-ESR, disease activity score in 28 joints with four variables including erythrocyte sedimentation rate; DAS28-CRP, disease activity score in 28 joints with four variables including C-reactive protein; SDAI, Simplified Disease Activity Index; CDAI, Clinical Disease Activity Index; HAQ-DI, Health Assessment Questionnaire Disability Index; mTSS, modified total Sharp score; JSN, joint space narrowing; JE, joint erosion; csDMARDs, conventional synthetic disease-modifying antirheumatic drugs; bDMARDs, biological disease-modifying antirheumatic drugs; tsDMARDs, target synthetic disease-modifying antirheumatic drugs; BMI, body mass index; ASMI, appendicular skeletal muscle mass index; BF%, percentage of body fat mass.

**Figure 3:**
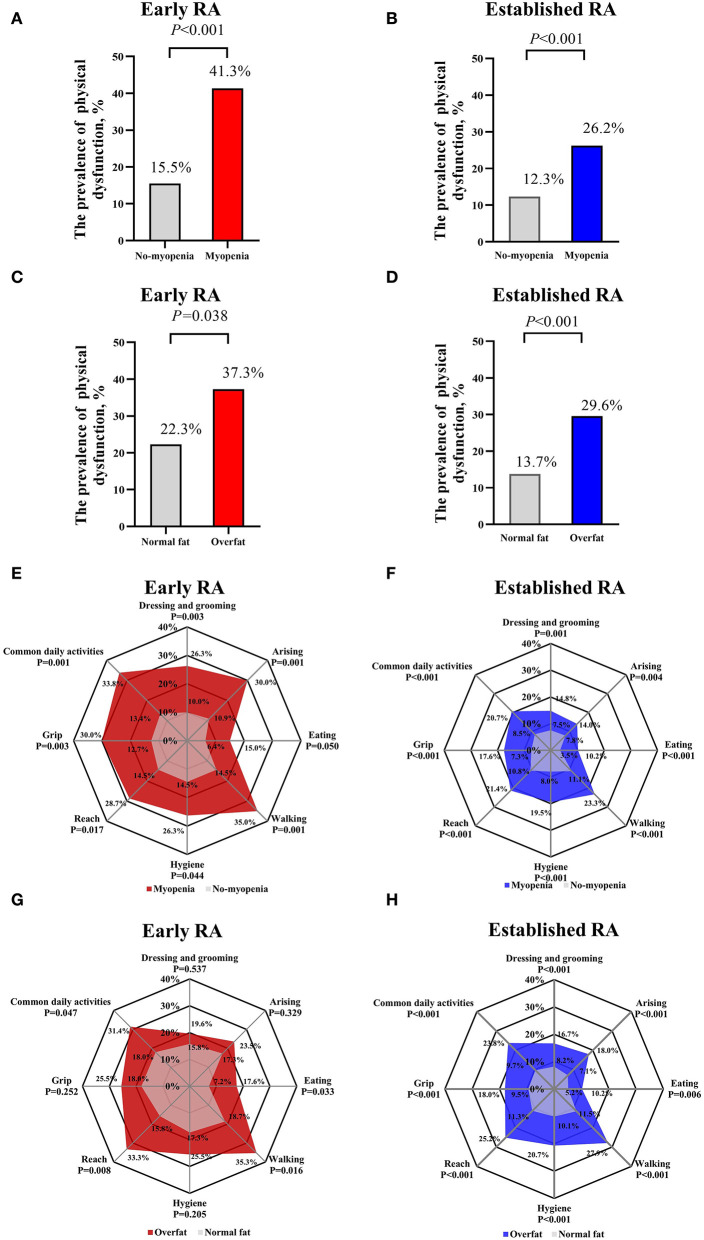
Comparisons of physical dysfunction between myopenia and overfat subgroups of patients with early and established RA. Comparisons of the prevalence of physical dysfunction (HAQ-DI > 1) between myopenia and overfat subgroups of patients with early **(A,C)** and established **(B,D)** RA; Comparisons of physical dysfunction of eight subdimensions (the scores of each subdimension > 1) between myopenia and overfat subgroups of patients with early **(E,G)** and established **(F,H)** RA. RA, rheumatoid arthritis; HAQ-DI, Health Assessment Questionnaire Disability Index.

On the other hand, patients with myopenia showed a higher prevalence of physical dysfunction than those without myopenia in both the patients with early RA (41.3 vs. 15.5%, *P* < 0.001) and established RA (26.2 vs. 12.3%, *P* < 0.001, Figures 3A,B). Meanwhile, the patients with overfat showed a higher prevalence of physical dysfunction than those without overfat in both patients with early RA (37.3 vs. 22.3%, *P* = 0.038) and established RA (29.6 vs.13.7%, *P* < 0.001, Figures 3C,D). The patients with myopenia in both the early RA and established RA groups had more physical dysfunction in all subdimensions than those without myopenia (Figures 3E,F). While the patients with overfat in the early RA group showed more physical dysfunction only in subdimensions of eating, walking, and common daily activities (Figure 3G), the patients with overfat in the established RA group showed more physical dysfunction in all subdimensions than those without (Figure 3H). Noteworthily, walking and common daily activities were the most involved subdimensions, and eating was the least involved subdimensions of physical dysfunction in the patients with myopenia or overfat in both the early and established RA groups.

### Associations of BC characteristics with physical dysfunction in patients with early and established RA

**Figure 4:**
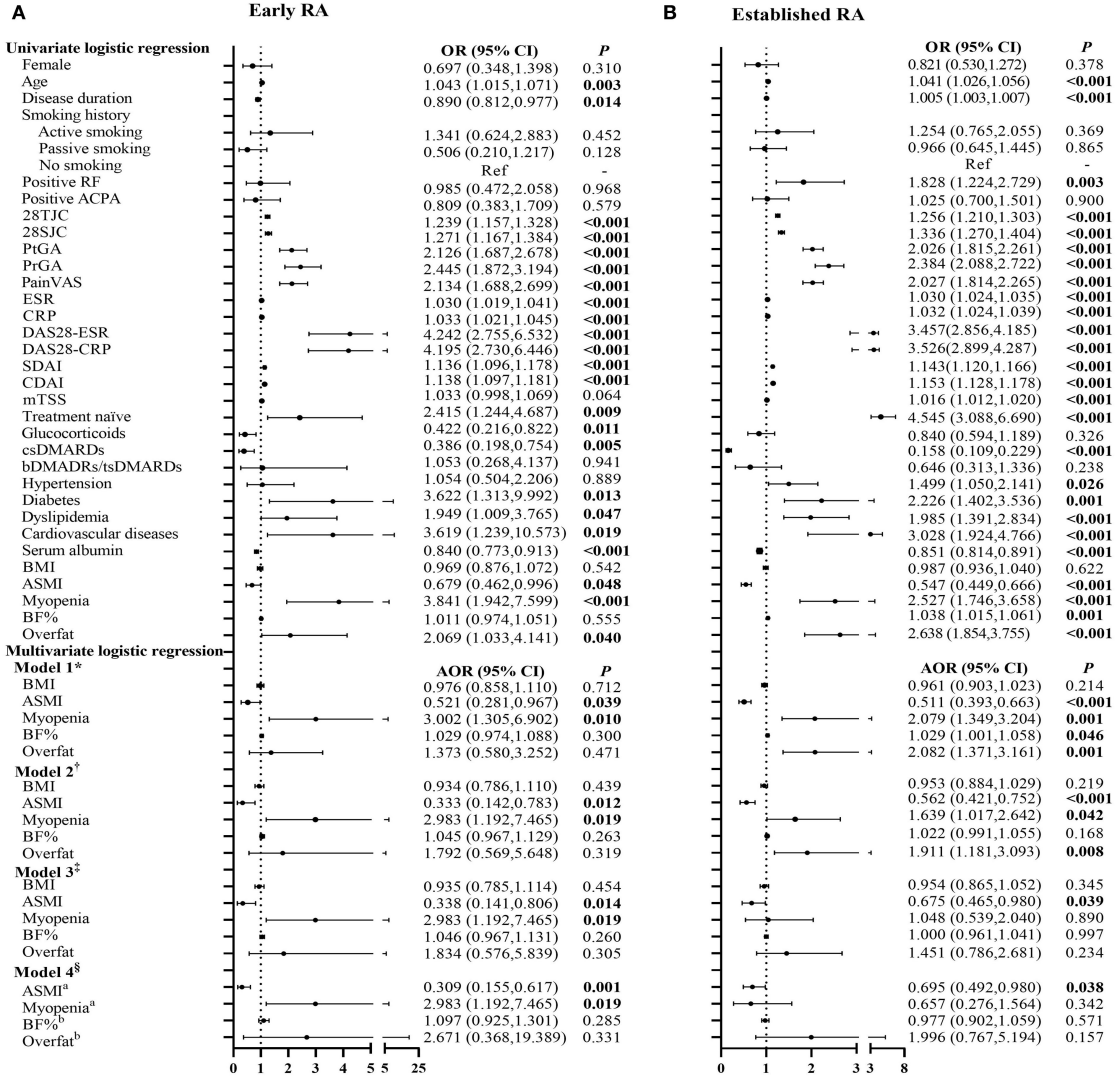
Logistic regression analysis of the relevant characteristics of physical dysfunction in patients with early and established RA. *Model 1 adjusted for gender, age, smoking history, disease duration, RF status, ACPA status, comorbidities, serum albumin, and previous treatment. ^†^Model 2 adjusted for Model 1+ DAS28-CRP (which was the most significantly associated with myopenia and overfat). ^‡^Model 3 adjusted for Model 2+ mTSS. ^§^Model 4 adjusted for Model 3+BMI+ ^a^BF%/^b^ASMI. OR, odds ratio in logistic regression; 95% CI, 95% confidence interval; RA, rheumatoid arthritis; RF, rheumatoid factor; ACPA, anti-cyclic citrullinated peptide antibody; 28TJC, 28-joint tender joint counts; 28SJC, 28-joint swollen joint counts; PtGA, patient global assessment of disease activity; PrGA, provider global assessment of disease activity; Pain VAS, pain visual analog scale; ESR, erythrocyte sedimentation rate; CRP, C-reactive protein; DAS28-ESR, disease activity score in 28 joints with four variables including erythrocyte sedimentation rate; DAS28-CRP, disease activity score in 28 joints with four variables including C-reactive protein; SDAI, Simplified Disease Activity Index; CDAI, Clinical Disease Activity Index; mTSS, modified total Sharp score; csDMARDs, conventional synthetic disease-modifying antirheumatic drugs; bDMARDs, biological disease-modifying anti-rheumatic drugs; tsDMARDs, target synthetic disease-modifying antirheumatic drugs; BMI, body mass index; ASMI, appendicular skeletal muscle mass index; BF%, percentage of body fat mass. **(A)** Early RA. **(B)** Established RA.

The association of BC characteristics with physical dysfunction in patients with early RA is shown in Figure 4A. In univariate analysis, ASMI was negatively associated and myopenia was positively associated with physical dysfunction, but no significant associations were found in BF% and overfat. After adjustment for all potential confounding factors by step (Model 1–Model 4), ASMI was still negatively [AOR 0.309, 95% CI (0.155–0.617)] and myopenia remained positively [AOR 2.983, 95% CI (1.192–7.465)] associated with physical dysfunction in the patients with early RA.

Figure 4B presents the relationship between BC characteristics with physical dysfunction in patients with established RA. In univariate analysis, ASMI was negatively associated and myopenia, BF%, and overfat were positively associated with physical dysfunction in patients with established RA. Multivariate analysis showed only a negative association of ASMI [AOR 0.695, 95% CI (0.492–0.980)] with physical dysfunction in the patients with established RA after adding mTSS for adjustment.

## Discussion

This cross-sectional study first compared BC characteristics among the matched patients with early and established RA as well as controls and revealed the early appearance of myopenia within 1 year of the RA disease duration (median 7 months) with a prevalence as high as that of established RA (41.3 vs. 50.4%). In the patients with early RA, the patients with myopenia showed a higher prevalence of physical dysfunction than those without myopenia (41.3 vs. 15.5%), and myopenia was independently associated with physical dysfunction (AOR = 2.983). These data indicated the importance of early detection of muscle involvement in the early stage of RA and imply the significance of early aggressive control of disease activity for the prevention of myopenia and physical dysfunction in patients with early RA.

Muscle mass loss has been recognized to be increasingly significant in patients with established RA (2–8). However, rare studies have investigated its presence in early RA, especially with a disease duration within 1 year (15). Book et al. found a lower appendicular lean mass in patients with early RA (*n* = 132, disease duration ≤ 12 months, mean 7 months) compared withmatched controls, but the proportion of patients with muscle mass loss was not reported (16). Müller et al. reported that patients with early RA within 1 year since disease onset (*n* = 91, mean 7 months) had a higher proportion of muscle mass loss than healthy age-matched controls (41.8 vs. 19.8%) (17). Other research studies recruited patients with early RA in a wide range of disease duration. Dao et al. found the prevalence of muscle mass loss was 18.1% in female patients with early RA (*n* = 105, disease duration ≤ 36 months) (18). Recently, Ekici et al. showed 31.5% of patients with newly diagnosed RA manifested loss of muscle mass (*n* = 91, disease duration 1–35 months) (19). Our large-scale study of 1,008 patients with RA confirmed the higher prevalence of myopenia in the patients with early RA (disease duration ≤ 12 months) compared with the age- and gender-matched controls (41.3 vs. 15.8%). Surprisingly, further comparison with the age- and gender-matched patients with established RA showed the prevalence of myopenia in the patients with early RA was even as high as that in the established stage (41.3 vs. 50.4%). Our data indicated early muscle involvement during the RA disease process.

The potential mechanisms of myopenia in RA may include increased pro-inflammatory cytokine production and chronic inflammation, changes in myokines (such as myostatin), changes in adipokines (such as adiponectin and leptin), chronic pain, physical limitation, and excessive resting energy expenditure (20–22). Myostatin negatively regulates skeletal muscles in elderly and animal models (22). Our previous study (23) reported that patients with RA showed a higher level of serum myostatin than healthy controls, but patients with RA with myopenia showed a lower level of serum myostatin than those without myopenia. Baker et al. (24) demonstrated that higher levels of adiponectin and leptin were independently associated with low lean mass in 419 patients from three RA cohorts. However, the link between inflammation and muscle mass loss has attracted more attention. It has been proved by *in vitro* and *in vivo* studies that pro-inflammatory cytokines could suppress the differentiation of myoblasts and induce muscle atrophy, such as TNF-α or IL-6 (25). Clinical studies confirmed that anti-TNF-α treatment by infliximab increased muscle mass in patients with Crohn's disease (26), while patients with RA gained appendicular lean mass after tocilizumab treatment by inhibiting IL-6 (27). A meta-analysis containing 3,140 RA subjects (most had a long-term disease duration of over 3 years) demonstrated that inflammation, such as higher DAS28, was notably associated with muscle mass loss (8). The hyperinflammatory state can occur in the early stage of RA. Einarsson et al. reported that patients with early RA (disease duration < 6 months) had a higher level of CRP and ESR and a higher HAQ score than those with established RA (disease duration > 24 months) (28). However, the developing time of muscle mass loss due to inflammation during the RA process remains unknown. In our study, the patients with early RA were characterized by higher disease activity scores as well as higher levels of ESR and CRP than the patients with established RA. In the patients with early RA, higher disease activity was independently associated with myopenia, indicating that a hyperinflammatory state may rapidly cause muscle mass loss within 1 year since RA onset. These data implied the significance of early aggressive control of inflammation and disease activity for the prevention of myopenia in patients with early RA.

Muscle mass loss may contribute to physical activity restriction, decreased strength, and deteriorated functional status in RA (29). Physical dysfunction is an important predictor of higher hospitalization, excessive healthcare costs, and premature mortality, in patients with established and early RA (30, 31). The causes of physical dysfunction in RA are multifactorial, and the most frequent factors include disease activity, joint pain and stiffness from synovitis, and joint deformity (32), which emphasize the importance of the RA disease course. In the early stage of RA, physical dysfunction is mainly caused by disease activity (specifically pain and synovitis), whereas joint damage becomes an important determinant in the long-term duration (33). A longitudinal study conducted over 20 years of 183 patients with early RA (disease duration ≤ 2 years) showed the disease activity score (estimated by Ritchie tender joint count, 44 swollen joint counts, and ESR) significantly contributed to the HAQ score, with a peak at baseline and then diminished continuously (contributions from 24 to 4%) (34). Moreover, the appendicular lean mass was found to be inversely associated with the HAQ score in patients with RA within 3 years since RA onset (18). Our previous studies found that patients with RA complicated with myopenia were characterized by high disease activity, physical dysfunction, and worsen joint destruction, and decreased muscle mass independently predicted the risk of 1-year aggravated joint destruction (3, 35). Myopenia can aggravate physical dysfunction as a mediator of age in elderly patients with RA (4). In this study, we found that myopenia was independently associated with physical dysfunction in early RA. Our data highlighted the important effect of muscle involvement on physical dysfunction, which implied the significance of muscle improvement for the prevention of physical dysfunction in patients with early RA. Leucine administration, progressive resistance training programs, the combination of protein supplementation plus muscle strengthening exercise, and the use of tocilizumab have been proven beneficial for gaining lean mass and physical function in the elderly (27, 36–38). These are worth further exploring in the future.

There were several limitations to our study. In this cross-sectional study, it was scientifically impropriety to determine the causality between myopenia and physical dysfunction in patients with early RA, owing to associated factors and outcome measurement being at the same timeframe. There was a lack of assessment of the physical activity level (e.g., sedentary lifestyle) and nutritional intake (e.g., protein intake), which were associated with muscle loss in RA. However, the level of serum albumin was used as an indicator of the nutritional state in our study. Although the level of serum albumin was negatively associated with physical dysfunction in patients with early RA by univariate analysis, myopenia remained positively associated with physical dysfunction after adjustment for all potential confounding factors including serum albumin (Figure 4). In this study, only 37.9% (25/66) of treatment-naive patients with early RA were found to have myopenia. Some medications for RA such as glucocorticoids at different dosage and durations might affect muscle and fat metabolism, although we have adjusted previous medications in multivariate logistic regression. Further multi-center prospective interventional studies with detailed medications would be needed in the future to investigate the development of myopenia and its clinical significance in patients with early RA.

## Conclusion

Myopenia appears commonly in the early stage of RA, and myopenia is associated with physical dysfunction in early RA. The early detection of muscle involvement is important in the early stage of RA, and the significance of muscle improvement for the prevention of physical dysfunction is worth further exploring in the future. Our study provides a new perspective on RA management.

## Data availability statement

The data analyzed in this study is subject to the following licenses/restrictions: the datasets used and/or analyzed during the current study are available from the corresponding author LD on reasonable request. Requests to access these datasets should be directed to LD, dailie@mail.sysu.edu.cn.

## Ethics statement

The studies involving human participants were reviewed and approved by the Ethics Boards of Sun Yat-Sen Memorial Hospital (SYSEC-KY-KS-012 and SYSEC-KY-KS-2020-208). The patients/participants provided their written informed consent to participate in this study.

## Author contributions

JP, Y-WZ, and Y-YZ participated in conceiving and designing the study, reading and analyzing documents, performing statistical analysis, and drafting the manuscript. J-ZL carried out the radiographic assessment and revised the manuscript. Z-HY carried out the radiographic assessment. TW, X-PZ, QZ, and H-WZ participated in data collection. X-LH and W-MC participated in the BC assessment of controls. J-DM and LD conceived and participated in the design of the study, read and analyzed documents, and edited the manuscript. All authors read and approved the final manuscript.

## Funding

This study was supported by the National Natural Science Foundation of China (82171780, 81971527, and 82101892), Guangzhou Municipal Science and Technology Project (202102010188), Basic and Applied Basic Research Foundation of Guangdong Province (2019A1515011928, 2020A1515110061, and 2022A1515010524), project funded by China Postdoctoral Science Foundation (2021M703722), Guangdong Medical Scientific Research Foundation (A2021065), and Fundamental Research Funds for the Central Universities, Sun Yat-sen University (22qntd3303).

## Conflict of interest

Authors X-LH and W-MC were employed by Shanghai Healthcare Co. Ltd. The remaining authors declare that the research was conducted in the absence of any commercial or financial relationships that could be construed as a potential conflict of interest.

## Publisher's note

All claims expressed in this article are solely those of the authors and do not necessarily represent those of their affiliated organizations, or those of the publisher, the editors and the reviewers. Any product that may be evaluated in this article, or claim that may be made by its manufacturer, is not guaranteed or endorsed by the publisher.

## References

[1] Smolen JS Aletaha D McInnes IB. Rheumatoid arthritis. Lancet. (2016) 388:2023–8. 10.1016/S0140-6736(16)30173-8 27156434

[2] Matsumoto Y Sugioka Y Tada M Okano T Mamoto K Inui K. Change in skeletal muscle mass is associated with lipid profiles in female rheumatoid arthritis patients -Tomorrow study. Clin Nutr. (2021) 40:4500–6. 10.1016/j.clnu.2020.12.028 33413913

[3] Lin JZ Liang JJ Ma JD Li QH Mo YQ Cheng WM. Myopenia is associated with joint damage in rheumatoid arthritis: a cross-sectional study. J Cachexia Sarcopenia Muscle. (2019) 10:355–67. 10.1002/jcsm.12381 30701694 PMC6463467

[4] Ma JD Chen CT Lin JZ Li QH Chen LF Xu YH. Muscle wasting, a neglected complication associated with physical dysfunction in elderly patients with rheumatoid arthritis: a cross-sectional observational study. Scand J Rheumatol. (2021) 50:280–9. 10.1080/03009742.2020.1842902 33554691

[5] Barone M Viggiani MT Anelli MG Fanizzi R Lorusso O Lopalco G. Sarcopenia in patients with rheumatic diseases: prevalence and associated risk factors. J Clin Med. (2018) 7:504. 10.3390/jcm7120504 30513782 PMC6306844

[6] Brance ML Di Gregorio S Pons-Estel BA Quagliato NJ Jorfen M Berbotto G. Prevalence of sarcopenia and whole-body composition in rheumatoid arthritis. J Clin Rheumatol. (2021) 27:S153–60. 10.1097/RHU.0000000000001549 32897991

[7] Fearon K Evans WJ Anker SD. Myopenia–a new universal term for muscle wasting. J Cachexia Sarcopenia Muscle. (2011) 2:1–3. 10.1007/s13539-011-0025-7 21475620 PMC3063883

[8] Li TH Chang YS Liu CW Su CF Tsai HC Tsao YP. The prevalence and risk factors of sarcopenia in rheumatoid arthritis patients: a systematic review and meta-regression analysis. Semin Arthritis Rheum. (2021) 51:236–45. 10.1016/j.semarthrit.2020.10.002 33385864

[9] Aletaha D Neogi T Silman AJ Funovits J Felson DT Bingham CO 3rd. 2010 Rheumatoid arthritis classification criteria: an American College of Rheumatology/European League Against Rheumatism collaborative initiative. Arthritis Rheum. (2010) 62:2569–81. 10.1002/art.27584 20872595

[10] Cush JJ. Rheumatoid arthritis: early diagnosis and treatment. Med Clin North Am. (2021) 105:355–5. 10.1016/j.mcna.2020.10.006 33589108

[11] Maska L Anderson J Michaud K. Measures of functional status and quality of life in rheumatoid arthritis: Health Assessment Questionnaire Disability Index (HAQ), Modified Health Assessment Questionnaire (MHAQ), Multidimensional Health Assessment Questionnaire (MDHAQ), Health Assessment Questionnaire II (HAQ-II), Improved Health Assessment Questionnaire (Improved HAQ), and Rheumatoid Arthritis Quality of Life (RAQoL). Arthritis Care Res. (2011) 63(Suppl.11):S4–13. 10.1002/acr.20620 22588760

[12] Baker JF George M Baker DG Toedter G Von Feldt JM Leonard MB. Associations between body mass, radiographic joint damage, adipokines and risk factors for bone loss in rheumatoid arthritis. Rheumatology. (2011) 50:2100–7. 10.1093/rheumatology/ker294 21890621

[13] Anker SD Morley JE von Haehling S. Welcome to the ICD-10 code for sarcopenia. J Cachexia Sarcopenia Muscle. (2016) 7:512–4. 10.1002/jcsm.12147 27891296 PMC5114626

[14] Oliveros E Somers VK Sochor O Goel K Lopez-Jimenez F. The concept of normal weight obesity. Prog Cardiovasc Dis. (2014) 56:426–33. 10.1016/j.pcad.2013.10.003 24438734

[15] Letarouilly JG Flipo RM Cortet B Tournadre A Paccou J. Body composition in patients with rheumatoid arthritis: a narrative literature review. Ther Adv Musculoskelet Dis. (2021) 13:1759720X211015006. 10.1177/1759720X211015006 34221129 PMC8221676

[16] Book C Karlsson MK Åkesson K Jacobsson LTH. Early rheumatoid arthritis and body composition. Rheumatology. (2009) 48:1128–32. 10.1093/rheumatology/kep165 19602478

[17] Müller R Kull M Põlluste K Valner A Lember M Kallikorm R. Factors associated with low lean mass in early rheumatoid arthritis: a cross- sectional study. Medicina. (2019) 55:730. 10.3390/medicina55110730 31717450 PMC6915666

[18] Dao HH Do QT Sakamoto J. Abnormal body composition phenotypes in Vietnamese women with early rheumatoid arthritis. Rheumatology. (2011) 50:1250–8. 10.1093/rheumatology/ker004 21292736

[19] Ekici R Erden A Güven SC Armagan B Özdemir B Karakaş Ö. Prevalence of sarcopenia and clinical implications in patients with newly diagnosed rheumatoid arthritis. Nutrition. (2021) 90:111353. 10.1016/j.nut.2021.111353 34192633

[20] Cohen S Nathan JA Goldberg AL. Muscle wasting in disease: molecular mechanisms and promising therapies. Nat Rev Drug Discov. (2015) 14:58–74. 10.1038/nrd4467 25549588

[21] Tuttle CSL Thang LAN Maier AB. Markers of inflammation and their association with muscle strength and mass: a systematic review and meta-analysis. Ageing Res Rev. (2020) 64:101185. 10.1016/j.arr.2020.101185 32992047

[22] Paris MT Bell KE Mourtzakis M. Myokines and adipokines in sarcopenia: understanding cross-talk between skeletal muscle and adipose tissue and the role of exercise. Curr Opin Pharmacol. (2020) 52:61–6. 10.1016/j.coph.2020.06.003 32668398

[23] Lin J-Z Ma J-D Yang L-J Zou Y-W Zhang X-P Pan J. Myokine myostatin is a novel predictor of one-year radiographic progression in patients with rheumatoid arthritis: a prospective cohort study. Front Immunol. (2022) 13:1005161. 10.3389/fimmu.2022.1005161 36330524 PMC9623067

[24] Baker JF Katz P Weber DR Gould P George MD Long J. Adipocytokines and associations with abnormal body composition in rheumatoid arthritis. Arthritis Care Res. (2021) 2021:acr.24790. 10.1002/acr.24790 34558809 PMC8942864

[25] Wang T. Searching for the link between inflammaging and sarcopenia. Ageing Res Rev. (2022) 77:101611. 10.1016/j.arr.2022.101611 35307560

[26] Subramaniam K Fallon K Ruut T Lane D McKay R Shadbolt B. Infliximab reverses inflammatory muscle wasting (sarcopenia) in Crohn's disease. Aliment Pharmacol Ther. (2015) 41:419–28. 10.1111/apt.13058 25580985

[27] Tournadre A Pereira B Dutheil F Giraud C Courteix D Sapin V. Changes in body composition and metabolic profile during interleukin 6 inhibition in rheumatoid arthritis. J Cachexia Sarcopenia Muscle. (2017) 8:639–46. 10.1002/jcsm.12189 28316139 PMC5566648

[28] Einarsson JT Willim M Ernestam S Saxne T Geborek P Kapetanovic MC. Prevalence of sustained remission in rheumatoid arthritis: impact of criteria sets and disease duration, a Nationwide Study in Sweden. Rheumatology. (2019) 58:227–36. 10.1093/rheumatology/key054 29538755

[29] Baker KJF Mostoufi-Moab S Long J Taratuta E Leonard MB Zemel B. Association of low muscle density with deteriorations in muscle strength and physical functioning in rheumatoid arthritis. Arthritis Care Res. (2021) 73:355–63. 10.1002/acr.24126 31841259 PMC7295665

[30] Hardy SE Kang Y Studenski SA Degenholtz HB. Ability to walk 1/4 mile predicts subsequent disability, mortality, and health care costs. J Gen Intern Med. (2011) 26:130–5. 10.1007/s11606-010-1543-2 20972641 PMC3019329

[31] Yelin E Trupin L Wong B Rush S. The impact of functional status and change in functional status on mortality over 18 years among persons with rheumatoid arthritis. J Rheumatol. (2002) 29:1851–7. 12233878

[32] Welsing PM van Gestel AM Swinkels HL Kiemeney LA van Riel PL. The relationship between disease activity, joint destruction, and functional capacity over the course of rheumatoid arthritis. Arthritis Rheum. (2001) 44:2009–17. 10.1002/1529-0131(200109)44:9<2009::AID-ART349>3.0.CO;2-L 11592361

[33] Hazes JM. Determinants of physical function in rheumatoid arthritis: association with the disease process. Rheumatology. (2003) 42(Suppl.2):ii17–21. 10.1093/rheumatology/keg328 12817091

[34] Kapetanovic MC Lindqvist E Nilsson JÅ Geborek P Saxne T Eberhardt K. Development of functional impairment and disability in rheumatoid arthritis patients followed for 20 years: relation to disease activity, joint damage, and comorbidity. Arthritis Care Res. (2015) 67:340–48. 10.1002/acr.22458 25186552

[35] Lin JZ Liu Y Ma JD Mo YQ Chen CT Chen LF. Reduced skeletal muscle independently predicts 1-year aggravated joint destruction in patients with rheumatoid arthritis. Ther Adv Musculoskelet Dis. (2020) 12:1759720X20946220. 10.1177/1759720X20946220 32922525 PMC7448126

[36] Martínez-Arnau FM Fonfría-Vivas R Buigues C Castillo Y Molina P Hoogland AJ. Effects of leucine administration in sarcopenia: a randomized and placebo-controlled clinical trial. Nutrients. (2020) 12:932. 10.3390/nu12040932 32230954 PMC7230494

[37] Hsu KJ Liao CD Tsai MW Chen CN. Effects of exercise and nutritional intervention on body composition, metabolic health, and physical performance in adults with sarcopenic obesity: a meta-analysis. Nutrients. (2019) 11:2163. 10.3390/nu11092163 31505890 PMC6770949

[38] Liao CD Chen HC Huang SW Liou TH. The role of muscle mass gain following protein supplementation plus exercise therapy in older adults with sarcopenia and frailty risks: a systematic review and meta-regression analysis of randomized trials. Nutrients. (2019) 11:1713. 10.3390/nu11081713 31349606 PMC6723070

