# Supplementary material for: Muscle mass loss is associated with physical dysfunction in patients with early rheumatoid arthritis
Source: Front Nutr. 2022 Nov 23;9:1007184. doi: 10.3389/fnut.2022.1007184 (PMC9727302; doi:10.3389/fnut.2022.1007184)
Supplement: Supplementary file 1 [file Table_1.docx]

**Supplementary Materials**

**Supplementary table captions**

**Supplementary Table S1 Comparisons of clinical characteristics between patients with early and established RA**

**Supplementary Table S2 Clinical characteristics of patients with overfat in early and established RA**

**Supplementary Table S3 Logistic regression analysis of the relevant characteristics with overfat in patiens with early and established RA**

**Supplementary Table S1 Comparisons of clinical characteristics between patients with early and established RA**

|  | All RA  (n=1008) | Early RA  (n = 190) | Established RA  (n =818) | *P^#^* |
| --- | --- | --- | --- | --- |
| Female, n (%) | 809 (80.3) | 136 (71.6) | 673 (82.3) | **0.001** |
| Age, years | 51.1±12.6 | 49.2±13.4 | 51.6±12.4 | **0.017** |
| Disease duration, month | 57 (21,120) | 7 (4,11) | 72 (36,127) | **<0.001** |
| Smoking history |  |  |  | **0.007** |
| Active smoking, n (%) | 158 (15.7) | 44 (23.2) | 114 (13.9) |  |
| Passive smoking, n (%) | 281 (27.9) | 49 (25.8) | 232 (28.4) |  |
| No smoking, n (%) | 569 (56.4) | 97 (51.1) | 472 (57.7) |  |
| Positive RF, n (%) | 687 (68.2) | 141 (74.2) | 546 (66.7) | **0.047** |
| Positive ACPA, n (%) | 724 (71.8) | 146 (76.8) | 578 (70.7) | 0.088 |
| Core disease activity indicators |  |  |  |  |
| 28TJC | 3 (1,7) | 4 (2,10) | 2 (0,7) | **<0.001** |
| 28SJC | 1 (0,4) | 3 (1,7) | 1 (0,4) | **<0.001** |
| PtGA | 3 (1,6) | 4 (2,6) | 3 (1,5) | **0.001** |
| PrGA | 3 (1,5) | 4 (2,6) | 3 (1,5) | **<0.001** |
| PainVAS | 3 (2,4) | 4 (2,5) | 2 (2,4) | **<0.001** |
| ESR, (mm/h) | 31 (16,57) | 41 (20,74) | 30 (16,53) | **0.002** |
| CRP, (mg/L) | 4.96 (3.28,19.98) | 9.69 (3.28,40.03) | 4.58 (3.28,17.75) | **<0.001** |
| DAS28-ESR | 3.07 (2.09,4.35) | 3.67 (2.70,4.85) | 2.92 (2.02,4.21) | **<0.001** |
| DAS28-CRP | 4.04 (2.83,5.47) | 4.76 (3.41,6.05) | 3.93 (2.73,5.37) | **<0.001** |
| SDAI | 13.10 (5.32,24.96) | 17.74 (8.34,34.30) | 12.25 (4.56,23.59) | **<0.001** |
| CDAI | 12 (5,22) | 16 (8,28) | 11 (4,21) | **<0.001** |
| Functional indicators |  |  |  |  |
| HAQ-DI | 0.25 (0.00,0.88) | 0.38 (0.00,1.12) | 0.25 (0.00,0.88) | **0.021** |
| Physical dysfunction, n (%) | 209 (20.7) | 50 (26.3) | 159 (19.4) | **0.035** |
| Radiographic assessment |  |  |  |  |
| mTSS | 9 (2,31) | 3 (0,7) | 14 (4,43) | **<0.001** |
| JSN | 2 (0,13) | 0 (0,2) | 5 (0,19) | **<0.001** |
| JE | 6 (2,18) | 2 (0,5) | 9 (3,23) | **<0.001** |
| Comorbidities |  |  |  |  |
| Hypertension, n (%) | 321 (31.8) | 48 (25.3) | 273 (33.4) | **0.031** |
| Diabetes, n (%) | 116 (11.5) | 17 (8.9) | 99 (12.1) | 0.220 |
| Dyslipidemia, n (%) | 326 (32.3) | 69 (36.3) | 257 (31.4) | 0.194 |
| Cardiovascular diseases, n (%) | 112 (11.1) | 15 (7.9) | 97 (11.9) | 0.117 |
| Nutritional indicator |  |  |  |  |
| Serum albumin, (g/L) | 34.5±4.6 | 33.9±4.8 | 34.7±4.5 | **0.024** |
| Previous medications |  |  |  |  |
| Treatment naïve, n (%) | 221 (21.9) | 66 (34.7) | 155 (18.9) | **<0.001** |
| Glucocorticoids, n (%) | 533 (52.9) | 98 (51.6) | 435 (53.2) | 0.691 |
| csDMARDs, n (%) | 713 (70.7) | 101 (53.2) | 612 (74.8) | **<0.001** |
| bDMADRs/tsDMARDs, n (%) | 76 (7.5) | 11 (5.8) | 65 (7.9) | 0.310 |

Values of continuous variables were presented as mean and standard deviation or median with interquartile range according to distributions, categorical variables were presented as numbers and percentages.

^#^Comparisons by Student’s *t*-test, Mann-Whitney U test, or chi-squared test. *P* value < 0.05 was defined as significant difference which was shown in bold.

RA, rheumatoid arthritis; RF, rheumatoid factor; ACPA, anti-cyclic citrullinated peptide antibody; 28TJC, 28-joint tender joint counts; 28SJC, 28-joint swollen joint counts; PtGA, patient global assessment of disease activity; PrGA, provider global assessment of disease activity; Pain VAS, pain visual analogue scale; ESR, erythrocyte sedimentation rate; CRP, C-reactive protein; DAS28-ESR, Disease Activity Score in 28 joints with four variables including erythrocyte sedimentation rate; DAS28-CRP, Disease Activity Score in 28 joints with four variables including C-reactive protein; SDAI, Simplified Disease Activity Index; CDAI, Clinical Disease Activity Index; HAQ-DI, Health Assessment Questionnaire Disability Index; mTSS, modified total Sharp score; JSN, joint space narrowing; JE, joint erosion; csDMARDs, conventional synthetic disease-modifying antirheumatic drugs; bDMARDs, biological disease-modifying anti-rheumatic drugs; tsDMARDs, target synthetic disease-modifying antirheumatic drugs

**Supplementary Table S2 Clinical characteristics of patients with overfat in early and established RA**

|  | Early RA | |  |  | | Established RA | |  |
| --- | --- | --- | --- | --- | --- | --- | --- | --- |
| Characteristics | Normal fat  (n=139) | Overfat  (n=51) | *P^#^* |  | Normal fat  (n=524) | | Overfat  (n=294) | *P**^#^* |
| Female, n (%) | 101 (72.7) | 35 (68.6) | 0.585 |  | 435 (83.0) | | 238 (81.0) | 0.459 |
| Age, years | 47.9±13.6 | 53.2±12.2 | **0.012** |  | 49.7±12.6 | | 54.9±11.3 | **<0.001** |
| Disease duration, month | 8 (4,11) | 7 (4,10) | 0.757 |  | 71 (36,120) | | 88 (42,155) | **<0.001** |
| Smoking history |  |  | 0.935 |  |  | |  | 0.545 |
| Active smoking, n (%) | 32 (23) | 12 (23.5) |  |  | 70 (13.4) | | 44 (15.0) |  |
| Passive smoking, n (%) | 35 (25.2) | 14 (27.5) |  |  | 155 (29.6) | | 77 (26.2) |  |
| No smoking, n (%) | 72 (51.8) | 25 (49) |  |  | 299 (57.1) | | 173 (58.8) |  |
| Positive RF, n (%) | 103 (74.1) | 38 (74.5) | 0.954 |  | 342 (65.3) | | 204 (69.4) | 0.230 |
| Positive ACPA, n (%) | 104 (74.8) | 42 (82.4) | 0.275 |  | 364 (69.5) | | 214 (72.8) | 0.316 |
| Core disease activity indicators |  |  |  |  |  | |  |  |
| 28TJC | 4 (1,9) | 5 (2,13) | 0.215 |  | 2 (0,5) | | 3 (1,9) | **0.001** |
| 28SJC | 3 (0,6) | 3 (1,9) | 0.206 |  | 1 (0,4) | | 2 (0,6) | **0.005** |
| PtGA | 4 (2,6) | 5 (2,7) | 0.041 |  | 3 (1,5) | | 4 (2,6) | **0.001** |
| PrGA | 4 (2,6) | 5 (2,7) | 0.071 |  | 3 (1,5) | | 4 (2,6) | **<0.001** |
| PainVAS | 4 (2,5) | 4 (2,6) | 0.055 |  | 2 (2,4) | | 3 (2,5) | **0.003** |
| ESR, (mm/h) | 43 (21,73) | 35 (17,79) | 0.740 |  | 27 (15,50) | | 36 (20,65.5) | **<0.001** |
| CRP, (mg/L) | 9.30 (3.32,39.24) | 10.21 (3.27,48.24) | 0.480 |  | 3.31 (3.19,13.64) | | 6.32 (3.33,24.38) | **<0.001** |
| DAS28-ESR | 4.74 (3.35,5.79) | 4.96 (3.49,6.40) | 0.247 |  | 3.83 (2.66,5.02) | | 4.42 (2.93,5.79) | **<0.001** |
| DAS28-CRP | 3.74 (2.68,4.79) | 3.61 (2.80,5.07) | 0.344 |  | 2.75 (1.92,3.934) | | 3.34 (2.26,4.65) | **<0.001** |
| SDAI | 16.48 (8.30,27.31) | 20.72 (10.31,36.79) | 0.131 |  | 10.84 (4.31,19.81) | | 15.03 (6.24,28.47) | **<0.001** |
| CDAI | 14 (7,24) | 18 (10,34) | 0.111 |  | 10 (4,18) | | 14 (5,25) | **<0.001** |
| Radiographic assessment |  |  |  |  |  | |  |  |
| mTSS | 2 (0,6) | 5 (1,8) | **0.019** |  | 12 (4,38) | | 17 (6,60) | **0.002** |
| JSN | 0 (0,1) | 0 (0,2) | 0.288 |  | 5 (0,16) | | 5 (1,23) | 0.100 |
| JE | 1 (0,4) | 4 (0,7) | **0.015** |  | 8 (3,21) | | 11 (4,31) | **0.002** |
| Comorbidities |  |  |  |  |  | |  |  |
| Hypertension, n (%) | 28 (20.1) | 20 (39.2) | **0.007** |  | 135 (25.8) | | 138 (46.9) | 0.084 |
| Diabetes, n (%) | 7 (5.0) | 10 (19.6) | **0.002** |  | 54 (10.3) | | 45 (15.3) | 0.409 |
| Dyslipidemia, n (%) | 47 (33.8) | 22 (43.1) | 0.236 |  | 139 (26.5) | | 118 (40.1) | **0.012** |
| Cardiovascular diseases, n (%) | 7 (5.0) | 8 (15.7) | **0.016** |  | 52 (9.9) | | 45 (15.3) | 0.327 |
| Nutritional indicator |  |  |  |  |  | |  |  |
| Serum albumin, (g/L) | 34.2±5.0 | 33.0±4.2 | 0.144 |  | 34.8±4.5 | | 34.5±4.6 | 0.313 |
| Previous medications |  |  |  |  |  | |  |  |
| Treatment naïve, n (%) | 46 (33.1) | 20 (39.2) | 0.432 |  | 90 (17.2) | | 65 (22.1) | 0.084 |
| Glucocorticoids, n (%) | 72 (51.8) | 26 (51) | 0.920 |  | 273 (52.1) | | 162 (55.1) | 0.409 |
| csDMARDs, n (%) | 78 (56.1) | 23 (45.1) | 0.177 |  | 407 (77.7) | | 205 (69.7) | **0.012** |
| bDMADRs/tsDMARDs, n (%) | 9 (6.5) | 2 (3.9) | 0.504 |  | 38 (7.3) | | 27 (9.2) | 0.327 |

Values of continuous variables were presented as mean and standard deviation or median with interquartile range according to distributions, categorical variables were presented as numbers and percentages.

^#^Comparisons by Student’s *t*-test, chi-squared test, or Mann-Whitney U test. *P* value <0.05 was defined as significant difference which was shown in bold.

RA, rheumatoid arthritis; RF, rheumatoid factor; ACPA, anti-cyclic citrullinated peptide antibody; 28TJC, 28-joint tender joint counts; 28SJC, 28-joint swollen joint counts; PtGA, patient global assessment of disease activity; PrGA, provider global assessment of disease activity; Pain VAS, pain visual analogue scale; ESR, erythrocyte sedimentation rate; CRP, C-reactive protein; DAS28-ESR, Disease Activity Score in 28 joints with four variables including erythrocyte sedimentation rate; DAS28-CRP, Disease Activity Score in 28 joints with four variables including C-reactive protein; SDAI, Simplified Disease Activity Index; CDAI, Clinical Disease Activity Index; mTSS, modified total Sharp score; JSN, joint space narrowing; JE, joint erosion; csDMARDs, conventional synthetic disease-modifying antirheumatic drugs; bDMARDs, biological disease-modifying anti-rheumatic drugs; tsDMARDs, target synthetic disease-modifying antirheumatic drugs

**Supplementary Table S3 Logistic regression analysis of the relevant characteristics with overfat in patients with early and established RA**

|  | Overfat in Early RA | |  | Overfat in Established RA | |
| --- | --- | --- | --- | --- | --- |
| Characteristics | **OR (95% CI)** | ***P*** |  | **OR (95% CI)** | ***P*** |
| **Univariate logistic regression** |  |  |  |  |  |
| Female | 0.823 (0.409,1.656) | 0.585 |  | 0.870 (0.601,1.259) | 0.459 |
| Age | 1.033 (1.007,1.060) | **0.013** |  | 1.037 (1.024,1.051) | **<0.001** |
| Disease duration | 0.987 (0.902,1.079) | 0.769 |  | 1.004 (1.002,1.006) | **<0.001** |
| Smoking history |  |  |  |  |  |
| Active smoking | 1.080 (0.483,2.414) | 0.851 |  | 1.086 (0.713,1.655) | 0.700 |
| Passive smoking | 1.152 (0.534,2.485) | 0.718 |  | 0.859 (0.616,1.196) | 0.367 |
| No smoking | Ref | - |  | Ref | - |
| Positive RF | 1.022 (0.490,2.131) | 0.954 |  | 1.206 (0.888,1.639) | 0.230 |
| Positive ACPA | 1.571 (0.695,3.550) | 0.278 |  | 1.176 (0.856,1.615) | 0.317 |
| BMI | 2.188 (1.709,2.801) | **<0.001** |  | 1.706 (1.580,1.843) | **<0.001** |
| 28TJC | 1.032 (0.982,1.085) | 0.213 |  | 1.046 (1.020,1.072) | **<0.001** |
| 28SJC | 1.035 (0.972,1.103) | 0.280 |  | 1.046 (1.014,1.080) | **0.005** |
| PtGA | 1.121 (0.993,1.267) | 0.065 |  | 1.095 (1.039,1.155) | **0.001** |
| PrGA | 1.109 (0.980,1.254) | 0.100 |  | 1.119 (1.059,1.183) | **<0.001** |
| PainVAS | 1.142 (0.995,1.310) | 0.059 |  | 1.097 (1.030,1.169) | **0.004** |
| ESR | 0.999 (0.990,1.008) | 0.837 |  | 1.008 (1.003,1.012) | **0.001** |
| CRP | 1.004 (0.996,1.012) | 0.280 |  | 1.009 (1.003,1.015) | **0.004** |
| DAS28-ESR | 1.118 (0.922,1.357) | 0.257 |  | 1.193 (1.096,1.298) | **<0.001** |
| DAS28-CRP | 1.145 (0.907,1.444) | 0.255 |  | 1.240 (1.120,1.374) | **<0.001** |
| SDAI | 1.016 (0.997,1.036) | 0.105 |  | 1.020 (1.010,1.030) | **<0.001** |
| CDAI | 1.018 (0.995,1.040) | 0.120 |  | 1.021 (1.010,1.032) | **<0.001** |
| mTSS | 1.019 (0.984,1.054) | 0.292 |  | 1.007 (1.004,1.011) | **<0.001** |
| Hypertension | 2.558(1.272,5.142) | **0.008** |  | 2.549(1.886,3.445) | **<0.001** |
| Diabetes | 4.599(1.646,12.851) | **0.004** |  | 1.573(1.029,2.405) | **0.036** |
| Dyslipidemia | 1.485(0.770,2.862) | 0.238 |  | 1.857(1.371,2.515) | **<0.001** |
| Cardiovascular diseases | 3.508(1.202,10.24) | **0.022** |  | 1.640(1.070,2.516) | **0.023** |
| Serum albumin | 0.950 (0.887,1.018) | 0.146 |  | 0.984 (0.953,1.015) | 0.313 |
| Treatment naïve | 1.304 (0.672,2.534) | 0.433 |  | 1.369 (0.958,1.956) | 0.085 |
| Glucocorticoids | 0.968 (0.509,1.839) | 0.920 |  | 1.128 (0.847,1.503) | 0.409 |
| csDMARDs | 0.642 (0.337,1.225) | 0.179 |  | 0.662 (0.479,0.914) | **0.012** |
| bDMADRs/tsDMARDs | 0.590 (0.123,2.825) | 0.509 |  | 1.293 (0.772,2.165) | 0.328 |
| **Multivariate logistic regression** |  |  |  |  |  |
| **Model 1**^*^ |  |  |  |  |  |
| DAS28-ESR | 0.996 (0.789,1.259) | 0.975 |  | 1.140 (1.027,1.266) | **0.014** |
| DAS28-CRP | 1.009(0.764,1.333) | 0.948 |  | 1.019(1.051,1.349) | **0.006** |
| SDAI | 1.004 (0.980,1.028) | 0.762 |  | 1.015 (1.003,1.027) | **0.012** |
| CDAI | 1.007 (0.981,1.034) | 0.585 |  | 1.015 (1.003,1.028) | **0.017** |
| **Model 2^†^** |  |  |  |  |  |
| DAS28-ESR | 0.996 (0.788,1.259) | 0.974 |  | 1.108 (0.978,1.256) | 0.107 |
| DAS28-CRP | 1.008 (0.763,1.332) | 0.957 |  | 1.192 (1.015,1.400) | **0.032** |
| SDAI | 1.004 (0.980,1.028) | 0.764 |  | 1.014 (1.000,1.029) | **0.046** |
| CDAI | 1.007 (0.981,1.034) | 0.587 |  | 1.014 (0.999,1.029) | 0.071 |
| **Model 3^‡^** |  |  |  |  |  |
| DAS28-ESR | 1.239 (0.358,4.290) | 0.736 |  | 1.027 (0.816,1.294) | 0.819 |
| DAS28-CRP | 1.068 (0.297,3.843) | 0.920 |  | 1.076 (0.798,1.451) | 0.630 |
| SDAI | 1.051 (0.893,1.237) | 0.551 |  | 1.012 (0.984,1.040) | 0.412 |
| CDAI | 1.144 (0.801,1.634) | 0.459 |  | 1.011 (0.982,1.041) | 0.452 |

^*^Model 1 Adjusted for gender, age, smoking history, disease duration, RF status, ACPA status, comorbidities, albumin and previous treatment

^†^Model 2 Adjusted for model 1 + mTSS

**^‡^**Model 3 Adjusted for model 2 + BMI, and ASMI

OR, odds ratio in logistic regression; 95% CI, 95% confidence interval

RA, rheumatoid arthritis; RF, rheumatoid factor; ACPA, anti-cyclic citrullinated peptide antibody; BMI, body mass index; 28TJC, 28-joint tender joint counts; 28SJC, 28-joint swollen joint counts; PtGA, patient global assessment of disease activity; PrGA, provider global assessment of disease activity; Pain VAS, pain visual analogue scale; ESR, erythrocyte sedimentation rate; CRP, C-reactive protein; DAS28-ESR, Disease Activity Score in 28 joints with four variables including erythrocyte sedimentation rate; DAS28-CRP, Disease Activity Score in 28 joints with four variables including C-reactive protein; SDAI, Simplified Disease Activity Index; CDAI, Clinical Disease Activity Index; mTSS, modified total Sharp score; csDMARDs, conventional synthetic disease-modifying antirheumatic drugs; bDMARDs, biological disease-modifying anti-rheumatic drugs; tsDMARDs, target synthetic disease-modifying antirheumatic drugs; ASMI, appendicular skeletal muscle mass index
